# Acute Stress Reaction in Combat: Emerging Evidence and Peer-Based Interventions

**DOI:** 10.1007/s11920-022-01335-2

**Published:** 2022-03-30

**Authors:** Amy B. Adler, Ian A. Gutierrez

**Affiliations:** Walter Reed Army Institute of Research, 503 Robert Grant Ave, Silver Spring, MD 20910 USA

**Keywords:** Combat experiences, Amygdala hijack, iCOVER, YaHaLOM, Peer support, High-risk occupations

## Abstract

**Purpose of Review:**

This paper highlights the topic of combat-related acute stress reactions (ASRs) in service members. Specifically, we contrast ASRs with related psychiatric conditions, report the estimated prevalence of ASRs for soldiers deployed to combat, and discuss how team members can effectively respond to these reactions.

**Recent Findings:**

Although not regarded as a clinical disorder, ASRs can have a significant impact on high-risk occupations like the military in which impaired functioning can imperil team members and others. Based on self-report, 17.2% of soldiers who have deployed to combat report having experienced a possible ASR. To our knowledge, this is the first such prevalence estimate.

**Summary:**

The prevalence of ASRs underscores the need for improved prevention, management, and recovery strategies. Peer-based intervention protocols such as iCOVER may provide a useful starting point to address ASRs in team members.

## Introduction

A small team of soldiers is on a mission to rescue stranded civilians and bring them out of a hostile country. The environment is harsh, the smells and sounds disorienting. Tension is high. As the team moves forward, they are ambushed by enemy combatants; they quickly take cover in an abandoned building. As they begin to respond to the threat, one soldier freezes in place, unable to return fire or follow directions. The situation is dangerous: the soldier is unable to act, and the team needs the soldier to snap back into action. What can the team members do?

Episodes like these, involving a service member frozen with fear, are not considered unusual in certain contexts like a combat deployment. In fact, these scenes are routinely depicted in all kinds of movies, ranging from *Saving Private Ryan* to *Avengers: Age of Ultron*. Although these moments are ubiquitous in fiction, it is only recently that there have been systematic attempts to understand and manage these moments of acute stress in real-world military operations.

In this paper, we review (1) acute stress reactions (ASRs) and how they differ from commonly understood traumatic responses and psychiatric diagnoses and conditions, (2) new evidence regarding self-reported ASR in soldiers experiencing combat, and (3) the development of peer-based interventions to help teams manage acute stress.

## Acute Stress

An ASR is defined in the International Classification of Disease, 11th edition (ICD 11; version 5/2021) as “the development of transient emotional, somatic, cognitive, or behavioural symptoms as a result of exposure to an event… of an extremely threatening or horrifying nature” [1]. These reactions can range from autonomic signs of anxiety, such as increased heart rate, sweating, and rapid breathing, to cognitive signs of anxiety, such as disorientation, non-responsiveness, or hyper alertness. The individual may be in a stupor or engage in overactivity. Regardless, these reactions are expected to “subside within a few days after the event or following removal from the threatening situation” [1].

While ASR appears in the ICD 11, it is not included in the Diagnostic and Statistical Manual of Mental Disorders, 5th Edition (DSM-5) [2]. It may be that ASR is excluded in the DSM-5 because it is not considered a disorder, but rather a natural disruption in functioning in the midst of extreme stress that should not be medicalized. In fact, ASR is defined in the ICD as “normal given the severity of the stressor” [1]. Whereas ASRs are fear-based responses to present real-world dangers, anxiety disorders manifest from an anticipation of possible future dangers owing to their occurrence in the past. The absence of a DSM-5 diagnosis for ASRs may also suggest that these symptoms are too transitory to be seen in a clinical setting or merit diagnosis. In other words, because service members may be in dire straits temporarily and then quickly recover, clinicians may not encounter patients with this particular symptom profile.

Although the DSM-5 does not include a diagnosis for ASR, it does include acute stress disorder (ASD) [2]. In contrast to ASR, ASD is characterized by intrusion symptoms, negative mood, dissociation, avoidance, and arousal, with symptoms defined as beginning after exposure to a traumatic stressor and lasting for at least 3 days and not longer than a month [2]. Posttraumatic stress disorder (PTSD) may be diagnosed for individuals reporting similar symptoms from four clusters (intrusion symptoms, avoidance, negative alterations in cognition and mood, and alterations in arousal and reactivity) lasting more than a month and leading to distress or functional impairment. Table 1 provides an overview of the features comprising ASR, ASD, and PTSD.

**Table 1:** Diagnostic features for acute stress reaction, acute stress disorder, and posttraumatic stress disorder

Diagnosis	Acute stress reaction (ASR)	Acute stress disorder (ASD)	Posttraumatic stress disorder (PTSD)
Description	• Autonomic signs of anxiety • Cognitive signs of being dazed or confused • Overactivity • Stupor	• Intrusion • Negative mood • Dissociation • Avoidance • Arousal	• Intrusion • Avoidance • Negative alterations in cognitions and mood • Marked alterations in arousal and anxiety
Symptom criteria	• Not specified • Regarded as a normal response to an extreme stressor	• At least 9 of 14 symptoms from any category	• At least 1 of 5 intrusion symptoms • 1 of 2 avoidance symptoms • 2 of 7 negative alterations in cognitions and mood symptoms • 2 of 6 symptoms of alterations in arousal and anxiety
Duration	Exposure to the severe stressor with reaction expected to subside within a few days	3 days to 1 month	More than 1 month
Distress or impairment in functioning	Not specified (but implied)	Yes	Yes

ASR is from the ICD 11; the details regarding ASD and PTSD are from the DSM-5. In the ICD 11, PTSD diagnosis requires symptoms of re-experiencing, avoidance, and perceptions of heightened current threat. ASD does not appear in ICD 11, but was in ICD 10

In light of their similar nomenclature, it is important for clinicians and researchers alike to distinguish between ASRs and ASD as different clinical phenomena. Whereas ASRs are conceptualized as an immediate and transitory set of physiological and psychological responses that occur during or in the immediate aftermath of a highly stressful or traumatic event and can be viewed as a normal response to such events, ASD constitutes a clinical disorder that comprises symptoms that persist days or weeks following a stressful event. Symptoms of ASD result in an impairment in normal functioning and bear a clinical resemblance to PTSD.

While an ASR may precede ASD or PTSD, the experience of an ASR does not guarantee the later presentation of ASD or PTSD symptoms, nor does the absence of an ASR in the midst of extreme stress preclude the presentation of ASD or PTSD in the weeks or months following the event. For example, although there is some relationship between peritraumatic distress and subsequent PTSD, this association wanes over time [3, 4]. Considering these different categorizations of trauma-related response together suggests that individuals may encounter a confluence of symptoms that can impair their functioning during a traumatic event; however, these symptoms may be fleeting and resolve themselves quickly. Once these kinds of symptoms are present for several days, individuals may be diagnosed with ASD or PTSD. Although early research suggested there may be a link between ASD and PTSD [5, 6], subsequent studies have not found this link [7•]. Importantly, however, there is no research that directly links ASR with subsequent disorders.

The closest evidence addressing the link between ASR and subsequent adjustment is Zahava Solomon’s longitudinal work with Israeli soldiers who participated in the 1982 Lebanon War [8, 9]. Solomon and colleagues compared soldiers who were referred by a battalion surgeon to psychiatric care for what they termed a combat stress reaction^1^–a shifting set of symptoms such as restlessness, withdrawal, and confusion–to soldiers who were not referred for a combat stress reaction. Soldiers with a combat stress reaction were at greater risk for subsequent development of PTSD 1 year [9] and 20 years later [8]. These analyses suggest a link between combat stress reaction and PTSD, but conclusions do not necessarily apply to an ASR because soldiers in this study were assessed after the traumatic event and after they had been referred for treatment. Consequently, it may be that soldiers who experienced a transitory ASR in the heat of the moment were not identified. Thus, the study may be assessing some other difficulty, such as ASD, rather than an ASR. Nevertheless, the Israeli study provides a unique look into the psychological stress experienced by soldiers soon after battle.

Taking these considerations into account, the transitory nature of ASRs suggests that an ASR diagnosis would have limited utility from a medical perspective: ASR is not a necessary prerequisite for the subsequent onset of ASD or PTSD, it is unlikely to be seen in a clinical setting, and ASD or PTSD warrant intervention regardless of whether the individual experienced an ASR at the time of the stressful event. However, an ASR can be meaningful and impactful in a high-stress occupational context, where its occurrence can place an individual at greater risk of injury, imperil the entire team if there are not enough individuals to sustain the safety of the group, or impede completion of a critical mission. Studies of high-demand professions have examined physiological and psychological distortions under stress that might illuminate the nature of acute stress. For example, there are several descriptive studies of police officers involved in shootings in which reactions such as visual or aural distortions, tunnel vision, temporal slowing, and memory distortion are reported (see [10] for a summary). Other studies of police-involved shootings focus on how stress impacts decision-making [11, 12], an important topic in its own right although one that is beyond the scope of the present paper.

It is also useful to distinguish panic attacks from ASRs. In both panic attacks and ASRs, the individual is overwhelmed by intense physiological and psychological stress, but there are important differences. Panic attacks are understood to occur within the context of a specified disorder (such as panic disorder), frequently present in the absence of a clearly identifiable external trigger, and are often recurrent. In contrast, ASRs appear to affect individuals regardless of their mental health history and are considered to be a normal response to a discrete and singular external threat. Symptom duration also appears to differ, with panic attacks resolving within minutes and an ASR resolving over a more variable time frame (from a few minutes to a few days). For individuals working in high-stress occupations, the potential for prolonged duration of symptoms in the context of an ASR is a critical concern.

## Recent Evidence of Prevalence During Combat

Despite the awareness that service members may be at risk for an ASR during combat, we are unaware of any previous work that has estimated the prevalence of service members who experience this kind of reaction. As an initial step toward quantifying and characterizing ASRs experienced during combat, our research team surveyed soldiers with combat experience. In the absence of a validated measure of ASR, we asked if soldiers had encountered a service member who was so mentally stressed during a significant combat-related event that the service member was unable to function for a period of time during the event or had some other difficulty in performance.

We surveyed two samples of soldiers who reported having deployed to combat and having experienced at least one combat-related event (176 soldiers in study 1 and 497 soldiers in study 2) and found that 51.7% and 42.4% of soldiers, respectively, reported witnessing unit members experience a possible ASR [13••]. The most common description endorsed was being unable to function and potentially increasing the risk to the team, with each of the descriptions endorsed by at least 19% of the sample that had observed a possible ASR in a teammate. These findings suggest that ASRs are not rare, that about half of soldiers experiencing high-risk combat environments may witness them, and that the reaction may present in a variety of ways. Working with researchers from the Israel Defense Forces, we found a roughly comparable rate of Israeli soldiers reporting that they had witnessed a teammate exhibit signs of an ASR (29% of 1,254 soldiers) [14•].

Building on this initial evidence, we then asked soldiers to report whether they had experienced an ASR themselves. In a pilot study, a small number of soldiers with previous combat deployment experience were asked to provide write-in descriptions of how unit members reacted to their possible ASR both at the time and afterwards. Responses are provided in Table 2. Themes describing actions taken during the combat-related event ranged from attempts to reassure the soldier to directing the soldier and focusing on the mission. Themes describing actions taken following the combat-related event included checking on the soldier, reassuring the soldier, ignoring the event, making a plan for the next time, and joking about it. Collectively, these themes reflect the fact that other unit members were largely aware of the event and that the need to keep the team focused on the mission was paramount. These themes also reflect what has been the lack of a systematic approach in handling an ASR.

**Table 2:** Self-reported unit response to soldier’s acute stress reaction

Theme	Response
**How did your unit members react at the time?**	
Offered reassurance	“They reassured me I was ok”
Assessed soldier’s status	“Asked if I was ok”
Directed soldier to action	“Pushed me and guided me to continue doing what I needed to”
	“Told me to shoot back”
	“It was loud a lot of yelling to direct what needed to happen”
Continued mission	“Others reinforced my position to provide covering fire”
	“Returned fire”
Did nothing	“They didn’t react. I overcame it w/o help.”
Other	“Uncertain”
**How did your unit members react afterwards?**	
Expressed concern	"Asked if I needed help"
	"They kept checking on me"
Made a plan	"Talked to me about what happened and how to deal with if next time"
Ignored it	"They didn't"
	"Was not discussed until years later"
	"Nothing"
Joked about it	"Laughed it off"
Offered reassurance	"Said it happens to everyone"
Other	"I don’t recall"
	"Exhausted"

Write-in responses on the survey questions completed by soldiers reporting yes or maybe/not sure to the statement: “During a significant combat-related event (such as a firefight or IED), I was so overwhelmed that I had difficulty functioning for a period of time”

These findings served as the basis for items developed for use in a subsequent survey with soldiers across a US Army Division. In all, 77.6% provided informed consent (7,403 out of 9,539 soldiers). Soldiers (*n* = 1,823) who reported having previously deployed to combat were asked to respond to the following statement describing a possible ASR: “During a significant combat-related event (such as a fire-fight or IED [improvised explosive device]), I was so overwhelmed that I had difficulty functioning for a period of time.” Of the 1,644 who provided a response to the question, 98 (6.0%) endorsed "yes," and 184 (11.2%) endorsed “maybe/not sure.” Taken together, 282 (17.2%) reported possibly experiencing an ASR, or more than 1 in 6.

In terms of rank, although a meaningful proportion of junior enlisted, non-commissioned officers (NCOs), and officers reported a possible ASR, there were significant differences, with 15.0% of junior-enlisted soldiers (E1-E4), 20.5% of NCOs, and 13.5% of officers reporting a possible ASR. In terms of gender, while there was not much difference between males (16.7%) and females (14.5%), 26.0% of those selecting “prefer not to answer” reported having a possible ASR.

**Fig. 1:**
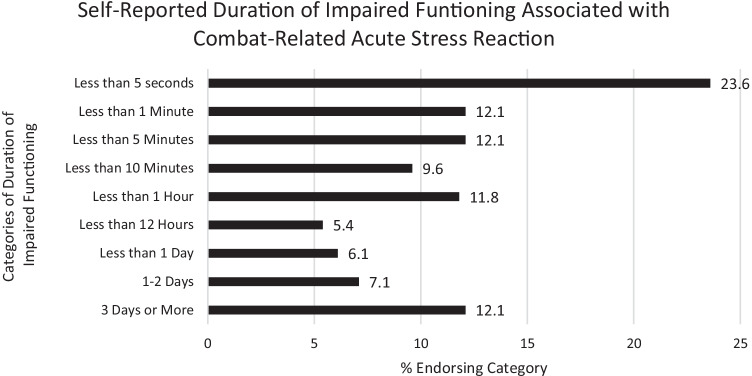
Self-reported duration of impaired functioning and combat-related acute stress reaction

We then asked those soldiers who experienced a possible ASR to estimate how long they had difficulty functioning. Frequencies are presented in Fig. 1. Note that while about half (47.8%) reported their difficulty in functioning was less than 5 min, just over half (52.2%) reported that they were impaired for more than 5 min, and nearly 1 in 5 (19.2%) reported that this impairment lasted more than a day. These data also suggest that 12.1% of soldiers reporting a possible ASR may be at risk of eventually qualifying for a diagnosis of ASD.

Soldiers who reported experiencing a possible ASR were then asked how their unit members responded *during* the period of time when they had difficulty functioning. These items were developed based on a thematic analysis of write-in responses from the previous survey. Approximately half (47.9%) reported unit members checked to see if they were okay; 25.9% reported unit members tried to reassure them; 19.5% reported that unit members tried to calm them down; 17.0% reported unit members assertively got their attention; 15.6% reported unit members told them what was happening; 11.3% reported unit members did nothing/ignored it; 11.0% reported unit members got them to safety; 7.8% reported unit members directed them to do a simple task; 7.1% reported unit members got someone else to help; 6.0% reported unit members yelled at them; 3.9% reported unit members shook, hit, or pushed them; and 26.6% reported they were not sure or did not remember.

Soldiers were then asked how their unit members reacted afterwards; survey items were derived from write-in responses from the previous survey. Nearly half (47.2%) reported unit members checked in on them, 27.0% reported unit members offered reassurance, 25.5% reported unit members expressed concern, 16.0% reported unit members did not react or ignored it, 12.4% reported unit members made a plan for what to do if it happened again, 14.2% reported unit members joked about it, and 4.3% reported unit members got upset with them.

We consider our estimates regarding the prevalence of a possible ASR to be relatively conservative because we included all those soldiers who reported deploying to a combat environment, without restricting our analysis to just those who reported at least one combat-related event in that environment. Thus, our denominator is relatively large, likely contributing to a smaller prevalence estimate. We chose this approach because our eight-item measure of possible combat events (e.g., “believed you would be seriously injured or killed,” “felt responsible for the death of a combatant”), while informative, was not exhaustive and therefore could not be used as a reliable filter. We did confirm, however, that soldiers endorsing a possible ASR reported nearly twice as many combat experiences as those who did not report a possible ASR, and our prevalence estimate increased to 22.8%—or more than 1 in 5—when we restricted our analysis to soldiers reporting at least one of these eight events. Regardless of which estimate is used, the frequency with which ASRs occur among soldiers during combat signals that there may be a significant impact on service member functioning and unit effectiveness.

Given that not all who deploy to combat will necessarily encounter an acute stress event, leaders and planners may want to take into account the kind of environment they expect deploying troops to face. For example, if service members were deploying on a humanitarian mission in which the environment was relatively safe and stable, a lower prevalence of ASR would be expected. In contrast, if service members were deploying to a conflict with a near-peer adversary [15], the prevalence of an ASR might be markedly higher. These estimates have practical utility for leaders so that they can anticipate momentary and prolonged gaps in functioning and introduce mitigating strategies accordingly. Behavioral health professionals can play a key role in reminding leaders to consider and plan for such eventualities. If, for example, a 100-person Army company were operating in a high-stress combat environment, it may help leaders to consider that 17 soldiers may experience an ASR, with 8 unable to function for what might be considered a meaningful amount of time.

We recognize that our data are limited by self-report, and there may be bias in retrospective reporting; however, we are unaware of any other realistic way to obtain prevalence statistics in light of the dangerous and unpredictable nature of a combat deployment. Previous research has also found that self-report of experiences during combat may be a valid approach to measurement [16]. Since these data are not reported through medical channels, there is also no way to review military records to validate these reactions. Instead, self-report is currently the most feasible and accurate method for obtaining prevalence estimates. Also, given that there may be perceptual distortions during an ASR, it is possible that soldier estimates of how long they felt their functioning was impeded may be inaccurate.

Despite these limitations, the results are based on a relatively robust sample and offer a window into understanding the experience of ASR from the soldier’s perspective. A substantial number of soldiers report what may be an ASR, this reaction is not purely fleeting for most, and their team members have an opportunity to respond in the moment. Although clinicians might ideally want to move a service member with an ASR to a relatively safer environment (while still following the principles of Proximity, Immediacy, Expectancy, and Simplicity; [17]), such an option may not be feasible when the reaction occurs in the middle of a mission where the need to return to functioning is paramount for everyone’s safety. Instead, it is particularly important to consider the role that team members can play in mitigating this threat to the individual and team.

## Peer-Based Intervention and Acute Stress Reaction

In 2014, the Israel Defense Forces launched *YaHaLOM* (an acronym that spells out a five-step intervention for peers to manage acute stress in team members: (1) Yetzirat kesher (Ya; [connect]); (2) Hadgashat (Ha; [emphasize]); (3) Levarer (L; [inquire]); (4) Vidu (O; [confirm]); and (5) Matan (M; [give])). This intervention involves getting the attention of the affected individual, reassuring them that they are not alone, asking them simple facts to trigger automatic cognitive processing, grounding them in the present moment, and directing them to engage in purposeful action. This simple and rapid technique was so well received that the Israel Defense Forces mandated *YaHaLOM* training for its service members. Initial survey results with 904 Israeli soldiers demonstrated that those who reported receiving *YaHaLOM* training reported more knowledge about and greater confidence in managing an ASR than those who did not report *YaHaLOM* training [18•]. Those reporting *YaHaLOM* training were also less likely to agree that soldiers who develop an ASR are weak and were more likely to agree that anyone could develop an ASR during combat [18•]. Survey results also demonstrated that including a video in training to depict the *YaHaLOM* steps was associated with better outcomes than training without a video [18•]. Training also moderated the relationship between witnessing an ASR and sub-clinical PTSD [14]. Specifically, soldiers indicating high levels of witnessing ASR in team members were less likely to endorse sub-clinical PTSD if they reported having been trained in *YaHaLOM* than if they reported they had not been trained [14]. Finally, case studies from the field demonstrated that the intervention could be implemented successfully in the real world [19••].

Building on the success of the Israeli program, the US Army adapted *YaHaLOM* into the six steps of iCOVER, which spells the six steps that comprise the intervention: Identify, Connect, Offer commitment, Verify facts, Establish order of events, and Request action. With the exception of including a new first step to identify individuals in need, these steps are essentially the same as in *YaHaLOM*. iCOVER training also differed from *YaHaLOM* in that iCOVER emphasized the rationale for each step and placed these steps into the larger context of field care management. A study with US service members demonstrated that iCOVER resulted in improved knowledge and attitudes compared to service members in a no-training comparison condition and that service members in the iCOVER condition followed more iCOVER steps during realistic training scenarios than untrained service members [20•]. A subsequent study with US soldiers preparing to deploy to combat demonstrated that even during this high-demand interval [21], iCOVER was associated with an increase in positive attitudes and confidence in unit members and leaders [22•]. In both of these iCOVER studies, service members gave the training exceptionally high ratings for utility and relevance.

Other national militaries are also adapting *YaHaLOM* and iCOVER for their own contexts. For example, Canada had developed has developed a protocol based on the three steps of “Calm, Connect and Coach”; Germany has developed BESSER; and Norway has developed ReSTART. Each country has created materials with its own organizational requirements in mind, and each is assessing perceptions of the training. The fact that these and other nations are adapting this training indicates that the gap in knowing how to manage ASR in team members is ubiquitous as is the interest in identifying potential solutions.

Besides peer-based interventions to support team members experiencing an ASR, training individuals to manage their own high stress may also be helpful. Although little is known about the ASR experience and whether an individual might be able to prevent its development, it may be useful for individuals to know that an ASR may resolve itself in less than 5 min so that they do not experience a spiraling of symptoms in response to anxiety about ASR. Techniques and mental skills used in evidence-based cognitive-behavioral therapies for managing panic and stress could also be adapted to potentially help service members prevent the onset of an ASR or mitigate its effects. These techniques include mindfulness practice for building awareness of rising stress levels in the moment, cognitive restructuring for managing fears and worries, grounding and deep breathing for reducing psychological and physical arousal, and self-talk for enhancing motivation and guiding completion of procedural tasks [23, 24]. It is also important to acknowledge that during an ASR, the individual may not be in a position to engage in their own self-care, underscoring the relevance of a peer-based approach.

## Conclusions

An ASR is characterized by a momentary and shifting set of psychological and physiological symptoms during a high-stress event. This reaction is distinguished by its impact on functioning. While the individual may recover quickly, for occupations that require individuals to operate under duress, even momentary breaks in functioning increase risk to the individual, teammates, and the mission.

While we have limited our focus for this paper to the prevalence of ASRs in the context of military combat events, the military is only one such occupation where ASRs might negatively impact team functioning. Other such occupational fields include policing, firefighting, and emergency medicine. Indeed, at the start of the COVID-19 pandemic in 2020, behavioral health providers supporting medical personnel in the civilian healthcare system reached out to the Army’s team responsible for iCOVER and asked for iCOVER to be adapted for medical staff. Medical teams were experiencing profound levels of acute stress as they responded to massive numbers of casualties at a relentless pace. iCOVER-Med was quickly developed and disseminated. While the steps remained the same, the rationale and examples were created for the medical context.

The fact that this request for iCOVER-Med spontaneously emerged from the civilian community suggests that acute stress may be a topic of interest not only for the military environment, but other high-risk occupations as well. Future research should study the degree to which ASRs impact functioning in a range of occupational settings and how it is typically addressed within teams. Theory and research on performance difficulties in high-stakes contexts such as sports and the stage may offer useful insight into conceptualizing ASRs, although such performance contexts entail being evaluated by others rather than exposure to horrific threat [25•]. In addition, future research should assess the impact of iCOVER training in a prospective intervention trial to determine if the training is effective, although anticipating the onset of an acutely stressful event is difficult, and following up with some high-risk teams over time may represent a feasibility challenge. Future research should also verify the estimates of duration in dysfunction associated with an ASR, perhaps through leveraging bodycam footage and audio. Finally, future research should examine the degree to which an ASR—and how it is managed by teammates—predicts subsequent mental health and the way in which facilitating a return to functioning may help offset feelings of frustration and inadequacy that could be harbored by individuals over time. By addressing these topics, individuals operating within high-risk occupations can be better prepared to help one another manage moments of extreme stress.

## Footnotes

1 The term “combat stress reaction” is generally a term used in military settings to refer to an ASR; however, sometimes the term “combat stress reaction” is used to encompass a wider range of symptoms than ASRs, including fatigue and misbehavior. In the present paper, we use the term ASR to focus attention on symptoms that occur in the heat of the moment, as described in the ICD 11, and by doing so broaden the relevance to other high-risk occupations such as policing, firefighting, and emergency medicine.
